# USP26 promotes anaplastic thyroid cancer progression by stabilizing TAZ

**DOI:** 10.1038/s41419-022-04781-1

**Published:** 2022-04-09

**Authors:** Jianing Tang, Yongwen Luo, Liang Xiao

**Affiliations:** 1 Department of Liver Surgery, Xiangya Hospital, Clinical Research Center for Breast Cancer Control and Prevention in Hunan Province, Central South University, Changsha, China; 2 Department of Urology, Zhongnan Hospital of Wuhan University, Wuhan, China

**Keywords:** Cancer, Oncogenes

## Abstract

Anaplastic thyroid cancer (ATC) is one of the most lethal and aggressive human malignancies, with no effective treatment currently available. The Hippo tumor suppressor pathway is highly conserved in mammals and plays an important role in carcinogenesis. TAZ is one of major key effectors of the Hippo pathway. However, the mechanism supporting abnormal TAZ expression in ATC remains to be characterized. In the present study, we identified USP26, a DUB enzyme in the ubiquitin-specific proteases family, as a bona fide deubiquitylase of TAZ in ATC. USP26 was shown to interact with, deubiquitylate, and stabilize TAZ in a deubiquitylation activity-dependent manner. USP26 depletion significantly decreased ATC cell proliferation, migration, and invasion. The effects induced by USP26 depletion could be rescued by further TAZ overexpression. Depletion of USP26 decreased the TAZ protein level and the expression of TAZ/TEAD target genes in ATC, including CTGF, ANKRD1, and CYR61. In general, our findings establish a previously undocumented catalytic role for USP26 as a deubiquitinating enzyme of TAZ and provides a possible target for the therapy of ATC.

## Introduction

Thyroid cancer is the most commonly diagnosed endocrine-related malignancy. Depending on the degree of differentiation, thyroid cancer has been traditionally categorized as either differentiated thyroid carcinoma (DTC) or undifferentiated/anaplastic thyroid carcinoma (ATC) [1]. DTC comprises of more than 90% of all thyroid cancers, including papillary and follicular carcinoma. This group of thyroid cancer exhibits good prognosis with >98% five-year survival [2]. ATC is a small subset of thyroid cancer. It is rare but extremely aggressive. Although ATC accounts for approximately 1–2% of thyroid cancers, it is responsible for half of thyroid cancer related deaths [3, 4]. Unlike normal follicular cells, ATC cells do not retain the biological functions such as iodine uptake and thyroglobulin synthesis, it is inherently resistant to both conventional chemotherapy and radioactive iodine. To date, there exist no effective therapies to cure or to prolong the survival of patients with ATC [5].

The Hippo pathway is an evolutionarily conserved pathway which was initially identified from Drosophila [6]. WW domain-containing transcription factor (WWTR1 or TAZ) and Yes-associated protein (YAP) are the two major downstream effectors. As transcriptional co-activators, YAP and TAZ mediate the biological functions of the Hippo pathway by regulating gene transcription [7]. The activity of YAP and TAZ can also be regulated in a Hippo-independent manner, which is composed of a kinase cascade: the upstream kinase MST1/2 promotes LATS1/2 phosphorylation and activation, leading to YAP/TAZ phosphorylation and inducing its cytoplasmic retention and subsequent β-TrCP-mediated proteasomal degradation. When Hippo signaling is off, YAP/TAZ enter the nucleus, and recruit other factors, such as TEAD and RUNX to activate genes involved in cell proliferation, migration, survival, and metabolism [8–10]. The dysregulation of Hippo pathway is thought to play a crucial role during tumor invasion and metastasis. YAP and TAZ are frequently activated in a variety of human malignancies. The activation of YAP/TAZ can promote cancer cell proliferation, metastasis, chemoresistance, and cancer stem cell-features, making them promising therapeutic targets in cancer [11]. While the underlying mechanisms regarding YAP/TAZ activation or overexpression in malignant tumors have not been well defined.

Accumulating studies indicate that the Hippo pathway is tightly modulated by the ubiquitin–proteasome system. A number of E3 ligases, such as PRAJA, ITCH, SIAH2, FBW7, and WWP1 are shown to play an essential role in controlling the abundance of several Hippo pathway components [12–14]. Deubiquitinases (DUBs) can reverse the ubiquitination of proteins by removing ubiquitin from the substrates. The DUBs in the human genome can be categorized into six families: ubiquitin COOH-terminal hydrolases (UCH), ubiquitin-specific proteases (USP), the JAB1/MPN/MOV34 family (JAMM), Josephins, ovarian tumor proteases (OTU), and motif interacting with ubiquitin-containing novel DUB family (MINDY) [15]. YOD1 was reported to induce LATS degradation and YAP/TAZ activation though de-ubiquitinating ITCH. The YOD1–ITCH–YAP/TAZ signaling axis would be a therapeutic target for liver cancer [16]. However, how DUBs regulate the Hippo signaling in ATC remains less well understood.

To investigate the involvement of DUBs in the Hippo pathway, we randomly selected 39 DUBs from a DUB siRNA library and conducted unbiased siRNA screening by monitoring the levels of TAZ. Among these, USP26 was observed to be the most potent DUB responsible for TAZ deubiquitination and stabilization in ATC. Furthermore, we found that USP26 promotes cell proliferation, migration and invasion through TAZ. Overall, our findings establish a previously undocumented catalytic role for USP26 as a deubiquitinating enzyme of TAZ and provides a possible target for the therapy of ATC.

## Materials and methods

### Cell culture

Human embryonic kidney HEK293T cell line and human anaplastic cancer cell lines (KHM-5M and CAL-62) were obtained from the Chinese Academy of Sciences (Shanghai, China). All cell lines were authenticated by the cell banks with short tandem repeat analysis. KHM-5M cells were cultured in RPMI 1640 Medium (42401, Life Technologies) containing 10% fetal bovine serum (FBS, Gibco) under 37 °C and 5% CO_2_ culture conditions. CAL-62 and HEK293T cells grown in Dulbecco’s Modified Eagle’s Medium (DMEM, HyClone) supplemented with 10% FBS under 37 °C and 5% CO_2_ culture conditions.

### Plasmids and siRNA

The The full-length and deletion mutant constructs of TAZ and USP26 were obtained from Hanbio Biotechnology Co. Ltd. (Shanghai, China). The HA-K6, HA-K11, HA-K27, HA-K29, HA-K33, HA-K48, HA-K63, and HA-Ub plasmids were acquired from Addgene. Small interfering RNAs targeting USP26 (siRNA-1: 5′-GCACAAGACUUCCGUUGGA-3′; 5′-AAACAGAUCUGGUUCACUU-3′) were synthesized by Genepharma (Shanghai, China).

### RNA extraction and qPCR analysis

Total RNA was extracted using MicroElute Total RNA Kit R6831-01 (Omega Bio-tek, Norcross, GA, USA) according to the manufacturer’s instructions. Reverse transcription was performed using HiScript III RT SuperMix (Vazyme, Nanjing, China). qRT-PCR was carried out was performed as we previously described [17]. The 36B4 gene was used as a reference for sample normalization.

### Western blot analysis

western-blot assays were performed according to the standard procedure to analyze protein expression in cells. Primary antibodies were used for assays: TAZ (Proteintech, 23306-1-AP), USP26 (sigma, SAB1300266), HA (Proteintech, 51064-2-AP), Myc (Proteintech, 60003-2-Ig), Flag (Proteintech, 66008-1-Ig), and GAPDH (Proteintech, 60004-1-Ig). Protein signals were visualized using an enhanced chemiluminescence (ECL) kit (Meilun, China) and detected by ChemiDoc XRS + Imaging System (Bio-Rad).

### Cell proliferation analysis

Cell proliferation was assessed by Cell Counting Kit-8 (CCK8) assay and EdU incorporation assay. Briefly, 2 × 10^3^ cells were seeded into duplicate wells of 96-well plates for CCK8 assay. Every 24 h, CCK8 solution reagent was added to each well and incubated for 1.5 h reaction at 37 °C. The absorbance at 450 nm was measured using a microplate reader. EdU incorporation assay was performed using EdU assay kit (Ribobio, Guangzhou, China) according to the manufacturer’s instructions.

### Clone formation assay

Cells were seeded in a 6-well plate at a low density (1000 cells per well). The cells were cultured in 2 ml of culture medium containing 10% FBS for two weeks, and the medium was refreshed every 3 days. The colonies were fixed with 4% paraformaldehyde and stained with crystal violet.

### Wound healing assay

Cells were seeded into 6-well plates until 100% confluence and we scratched cell layer with a sterile tip. Cells were maintained in the medium containing 1% FBS. The cells were captured at indicated time points.

### Transwell assay

Cell invasion capacity was assessed using 8 μm pore polycarbonate membrane transwell plates (Corning, USA). Briefly, 5 × 10^5^ cells were suspended without serum and were seeded into the upper chambers precoated with matrigel (BD Biocoat, USA). The bottom chambers were filled with 600 μl complete medium. After 24 h, the cells on the bottom side of the pore membrane were fixed and stained with crystal violet.

### In vivo tumorigenesis and metastasis assay

BALB/c nude mice aged 4 weeks were obtained from Beijing HFK Bioscience Co., Ltd. in Beijing, China. For in vivo tumorigenic experiment, 1 × 10^6^ CAL-62 cells were injected to the right dorsal flank of each mouse. Tumor sizes were measured every 3 days until the end of the experiment. For in vivo metastasis assays, 0.5 × 10^6^ CAL-62 cells were intravenously injected into each mouse through the tail vein. The lungs were harvested 4 weeks after injection. The mice were maintained in a temperature and humidity‐controlled and specific pathogen‐free environment in the laboratory animal facility of Xiangya Hospital of Central South University. The experiments were performed under the protocols approved by ethnic committee of Xiangya Hospital of Central South University.

### Luciferase assay

The TAZ/TEAD luciferase reporter plasmid, Renilla plasmid and USP26 siRNAs were transfected together the into CAL-62 cells. Twenty-four hours after transfection, luciferase activity was detected using the Dual-Luciferase Reporter kit (Promega, Germany).

### Co-immunoprecipitation (co-IP) assay

Cells were lysed with co-IP lysis buffer (Meilun, China). containing a cocktail of protease inhibitors. The total cell lysis were precleared with rabbit IgG for 2 h and subsequently immunoprecipitated with indicated antibody and Protein A/G PLUS-Agarose beads (Santa Cruz) at 4 °C overnight. After washing the immunocomplexes three times with lysis buffer, the immunoprecipitated proteins were collected for Western blot analysis.

### GST pulldown assays

Two microgram recombinant GST or GST-TAZ was incubated with 2 μg of recombinant USP26 and immobilized glutathione beads (Samgon Biotech, China) in binding buffer (0.5 mM EDTA, 10 mM HEPES pH 7.5, 0.1% NP-40, 50 mM NaCl, and 0.5 mM DTT) at 4 °C for 2 h. Then the beads were washed with GST binding buffer. The bound proteins were collected for western blot analysis.

### In vivo deubiquitination assay

HEK293T cells were co-transfected with HA-Ub, Flag-TAZ, Myc-USP26, or Myc-USP26^C304S^ plasmid as indicated using Lipofectamine 2000 for 24 h. After incubation with 10 μM MG132 (MCE) for 6 h, HA-ubiquitinated TAZ was immunoprecipitated with anti-Flag antibody followed by western blot analysis.

### In vitro deubiquitination assay

For the in vitro deubiquitination assay, Flag-TAZ and HA-Ub plasmids were co-expressed in HEK293T cells and purified using anti-FLAG M2 affinity gels (Sigma Aldrich). The ubiquitinated TAZ proteins were incubated with bacterially purified GST or GST-USP26 proteins in deubiquitination buffer (50 mM NaCl, 50 mM Tris-HCl, 10 mM dithiothreitol, 1 mM EDTA, 5% glycerol, and pH 8.0) at 37 °C for 2 h. And deubiquitination was analyzed by western blot.

### Statistical analysis

Statistical analysis was performed using Prism 7.0 (GraphPad, USA) with two-tailed student’s t-test or one-way ANOVA. Multiple comparison was carried out using Bonferroni’s adjustment. The relation between USP26 expression and clinicopathological characteristics was analyzed by Pearson *χ*^2^ test. Data with *P*-value <0.05 was considered as statistically significant.

## Results

### USP26 depletion inhibits Hippo signaling pathway activity

**Fig. 1:**
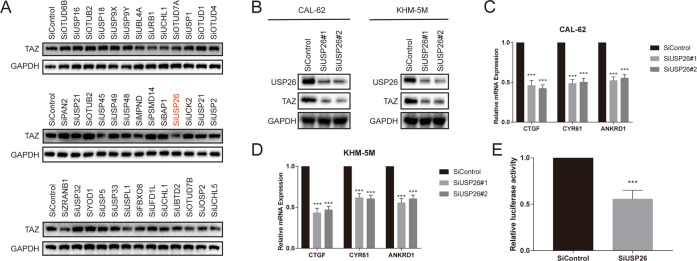
USP26 depletion decreases Hippo signaling activity in ATC cells. **A** The siRNAs specific to each deubiquitinating enzyme were transfected into CAL62 cells. After 48 h, cells were lysed and the TAZ protein level was analyzed by Western blot. **B** USP26 depletion decreased TAZ protein level. **C**, **D** USP26 depletion decreased the expression of TAZ target genes using two different siRNA oligos. **E** USP26 depletion decreased TAZ-luciferase activity. CAL62 cells were transfected with Si USP26 or SiControl together with TAZ/TEAD-luciferase reporter plasmid. Luciferase activity was measured 48 h after transfection. *P value < 0.05; **P value < 0.01; ***P value < 0.001.

To systematically determine the involvement of DUBs in Hippo signaling pathway, we randomly selected 39 DUBs from a DUB siRNA library and conducted unbiased siRNA screening by monitoring the levels of TAZ. Among these DUBs, USP26 was observed to potentially deubiquitinate and stabilize TAZ as its siRNA-mediated depletion markedly reduced TAZ abundance (Fig. 1A). We further confirmed that USP26 depletion decreased TAZ protein level in CAL-62 and KHM-5M cells by using two non-overlapping siRNAs separately (Fig. 1B). Consistently, the transcript levels of YAP/TAZ target genes (CTGF, CYR61, and ANKRD1) were significantly decreased in cells depleted with USP26 (Fig. 1C, D). And the knockdown of USP26 reduced YAP/TAZ reporter activity as well (Fig. 1E). These results indicate that USP26 is a regulator of Hippo signaling pathway through YAP/TAZ.

### USP26 interacts with TAZ

**Fig. 2:**
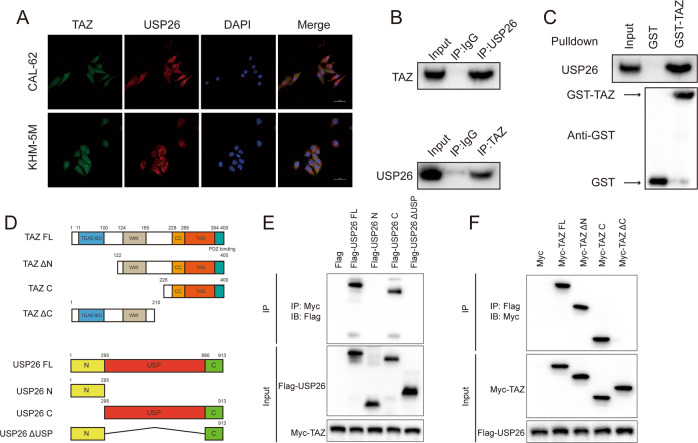
USP26 associates with TAZ and increases its stability. **A** An immunofluorescence assay demonstrated that USP26 and TAZ at least partially colocalized in CAL62 and KHM-5M cells (×400 magnification). **B** Co-IP assay revealed an association between endogenous USP26 and TAZ in CAL62 cells. CAL62 cells were harvested with RIPA lysis buffer. Co-IP was performed using antibody as indicated. **C** Recombinant USP26 were incubated with GST-TAZ or GST protein. The interacted USP26 was detected by western blot. **D** USP26 and TAZ domain structure and deletion mutants used in the study. **E** The USP domain of USP26 interacted with TAZ. HEK293 cells were transfected with 2 µg Myc-TAZ together with Flag-USP26 full length or mutants. After 24 h, cells were harvested with NP-40 lysis buffer. Co-IP was performed using Myc antibody. The possible interacted USP26 domains were detected by Flag antibody. **F** TAZ interacted with USP26 through its C terminal. HEK293 cells were transfected with 2 µg Flag-USP26 together with Myc-TAZ full length or mutants. After 24 h, cells were harvested with NP-40 lysis buffer. Co-IP was performed using Flag antibody. The possible interacted TAZ domains were detected by Myc antibody.

Results of immunostaining indicated that USP26 and TAZ at least partially co-localized in CAL-62 and KHM-5M cells (Fig. 2A). Co-IP of the endogenous proteins from CAL-62 cells showed that USP26 could interact with TAZ, supporting for the functional cooperation of USP26 and TAZ (Fig. 2B). And we observed identical binding between the constitutively active form of TAZ (S89A) and USP26 (Fig. S1A). Further GST-pulldown assay verified the direct interaction between USP26 and TAZ (Fig. 2C). In order to investigate the interaction domain between the two proteins, we generated a series of truncated TAZ and USP26 constructs (Fig. 2D). Co-IP assays showed that the USP domain of USP26 was in charge of its interaction with TAZ, while the C-terminal region of TAZ was responsible to associate with USP26 (Fig. 2E, F).

### USP26 modulates TAZ stability in a DUB-dependent manner

**Fig. 3:**
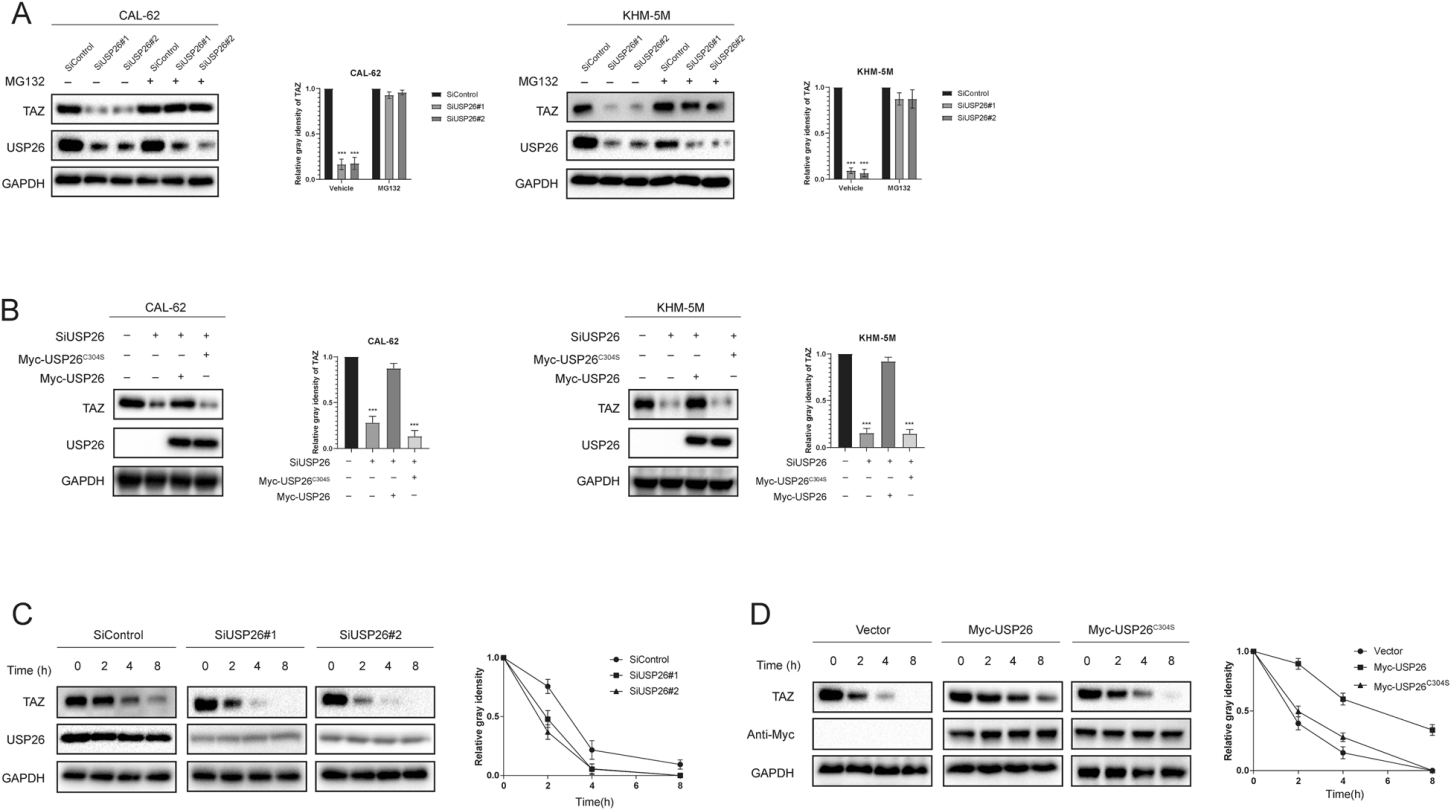
USP26 increases TAZ stability. **A** In the presence of the proteasome inhibitor MG132, depletion of USP26 did not further decrease the TAZ protein level. ATC cells were transfected with siUSP26 or siControl. After 48 h, cells were treated with 10 µM MG132/vehicle for 6 h, cell lysates were prepared for western blot analysis. **B** ATC cells were transfected with USP26 (wild type or C304S) together with USP26 siRNA. The TAZ level were measured. **C** USP26 depletion decreased TAZ half-life in CAL-62 cells. CAL-62 cells were transfected with siUSP26 or siControl. After 48 h, cells were treated with 100 µM cycloheximide/vehicle for indicated times. Cell lysates were prepared for western blot analysis. **D** USP26^C304S^ did not increase TAZ half-life in HEK293 cells. HEK293 cells were transfected with Myc-tag, Myc-USP26 or Myc-USP26^C304S^ plasmids. After 24 h, cells were treated with 100 µM cycloheximide/vehicle for indicated times. Cell lysates were prepared for Western blot analysis.

There are two possible explanation for USP26 in regulating TAZ protein level, which could be either transcriptional regulation or posttranslational regulation. Results of qPCR analysis indicated that TAZ mRNA level was not changed upon USP26 depletion (Fig. 3A). As shown in Fig. 3B, knockdown of USP26 dramatically decreased TAZ protein level, while the proteasome inhibitor MG132 could abolish the inhibition effect of TAZ protein induced by USP26 depletion. USP26 regulates TAZ in a DUB-dependent manner as the catalytically inactive mutant C304S lost its ability to upregulate TAZ (Fig. 3C). To prove that USP26 modulates TAZ stability, we then treated cells with the protein synthesis inhibitor cycloheximide (CHX). The half-life of TAZ was significantly shortened in cells depleted of USP26. On the other hand, overexpression of the wild type USP26, but not C304S, prolonged the half-life of TAZ (Fig. 3D). These results demonstrate that USP26 increases TAZ stability in a DUB-dependent manner.

### USP26 deubiquitylates TAZ

**Fig. 4:**
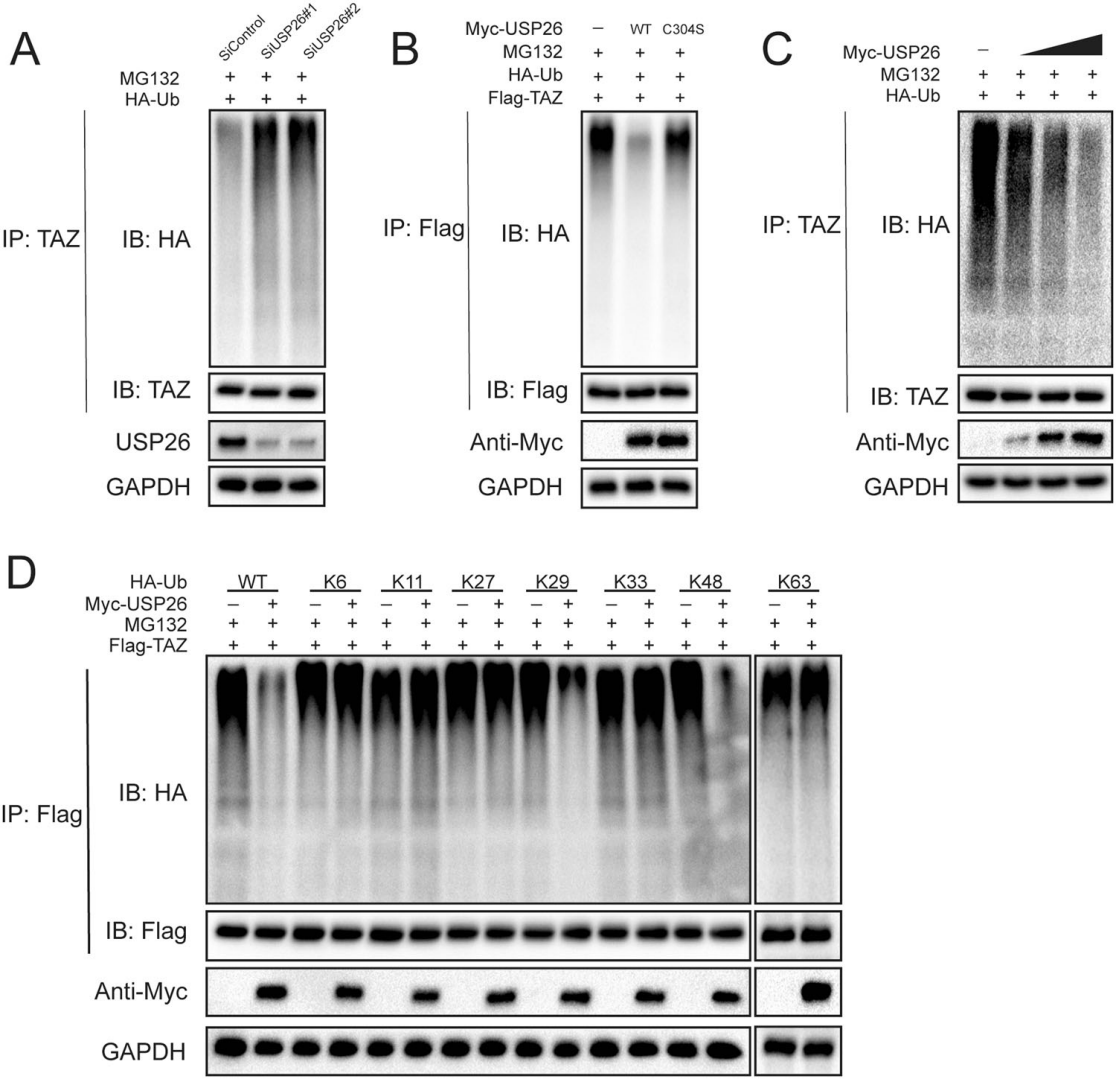
USP26 deubiquitylates TAZ. **A** CAL-62 cells transfected with the indicated siRNA were treated with MG132 for 6 h before collection. TAZ was immunoprecipitated with anti-TAZ and immunoblotted with anti-HA. **B** Immunoblotting to detect the ubiquitination of TAZ in HEK293 cells co-transfected with Flag-TAZ, HA-Ubiquitin and Myc-USP26 (wild type or C304S). **C** USP26 removed the ubiquitin chain of TAZ in a dose-dependent manner. **D** HA-WT, K6, K11, K27, K29, K33, K48, or K63 Ub was co-transfected with Flag-TAZ and Myc-USP26 into HEK293 cells. After treatment with 10 μM MG132 for 6 h, cell lysates were subjected to ubiquitination assay and the ubiquitination level of TAZ was detected by HA antibody.

As USP26 is member of the USP family of DUBs family, we went on to determine the possibility that USP26 deubiquitylates TAZ. The level of polyubiquitin chains on TAZ was significantly increased in CAL-62 cells depleted with USP26 (Fig. 4A). Conversely, ectopic expression of wild type USP26, but not C304S, reduced TAZ ubiquitylation in cells (Fig. 4B). We also observed identical de-ubiquitination between the constitutively active form of TAZ and the wild type TAZ (Fig. S1B). In vitro ubiquitylation assay further confirmed that USP26 directly removed the ubiquitin chain from TAZ (Fig. S2). Supporting our former conclusion that the catalytical activity is essential for USP26 to increase TAZ stability. In addition, we observed that USP26 deubiquitylated TAZ in a dose-dependent manner (Fig. 4C). It is well known that ubiquitin has several lysine residues (Ub-K6, Ub-K11, Ub-K27, Ub-K29, Ub-K33, Ub-K48, and Ub-K63), which can be used to form distinct linkage types of ubiquitin chains and perform different cellular functions. To identify which type of ubiquitin chain of TAZ was affected by USP26, we cotransfected HEK293T cells with TAZ, USP26, and HA-tagged WT ubiquitin and K48-specific, K63-specific, K6-specific, K11-specific, K27-specific, K29-specific, or K33-specific ubiquitin. Our results indicated that USP26 could efficiently remove K29- and K48-linked ubiquitin chain from TAZ (Fig. 4D). Taken together, USP26 is a specific DUB responsible for TAZ deubiquitination and stabilization.

### USP26 promotes ATC progression via TAZ

**Fig. 5:**
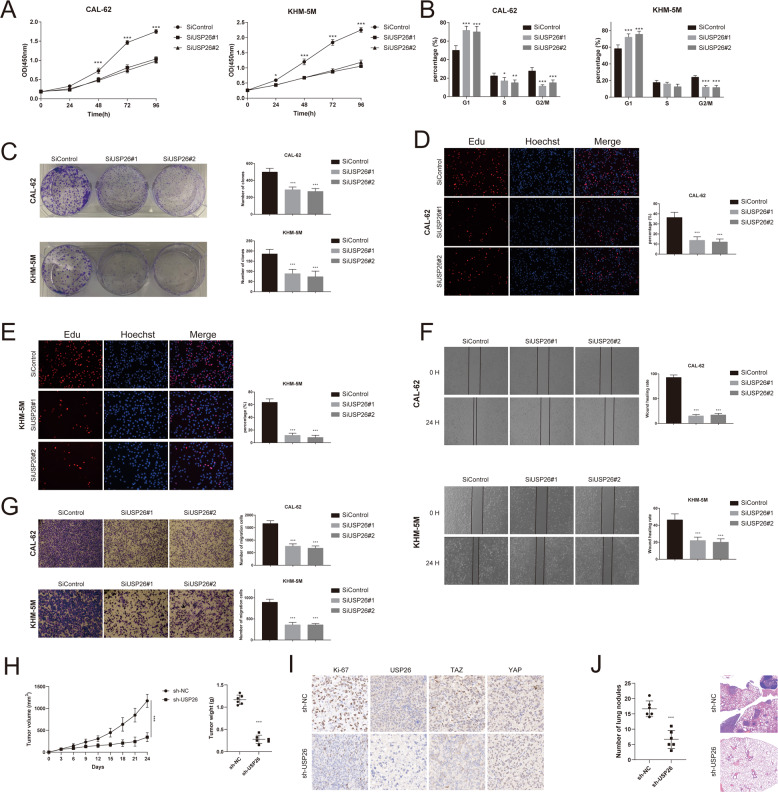
USP26 depletion inhibits ATC cell proliferation, migration andinvasion. **A** USP26 depletion inhibited cell proliferation in breast cancer cells. **B** USP26 depletion induced G1 cell cycle arrest in ATC cells. **C** USP26 depletion decreased clone formation capability of ATC cells. **D**, **E** Representative images of EdU assay of ATC cells. **F** Wound-healing assay of ATC cells. **G** Tranwell invasion assay of ATC cells. **H** USP26 depletion inhibits the tumor growth in vivo. CAL-62 cells were stably transfected with lentivirus carrying a scrambled shRNA or USP26 shRNA. 1 × 10^6^ CAL62 cells were injected to the right dorsal flank of each mouse (*n* = 6). Tumor sizes were measured every 3 days until the end of the experiment. **I** Representative images of immunohistochemical staining for Ki67, USP26, and TAZ. **J** USP26 depletion suppressed the lung metastasis of ATC in mice. 0.5 × 10^6^ ATC cells were intravenously injected into each mouse through the tail vein. The lungs were harvested 4 weeks after injection. *P value < 0.05; **P value < 0.01; ***P value < 0.001.

**Fig. 6:**
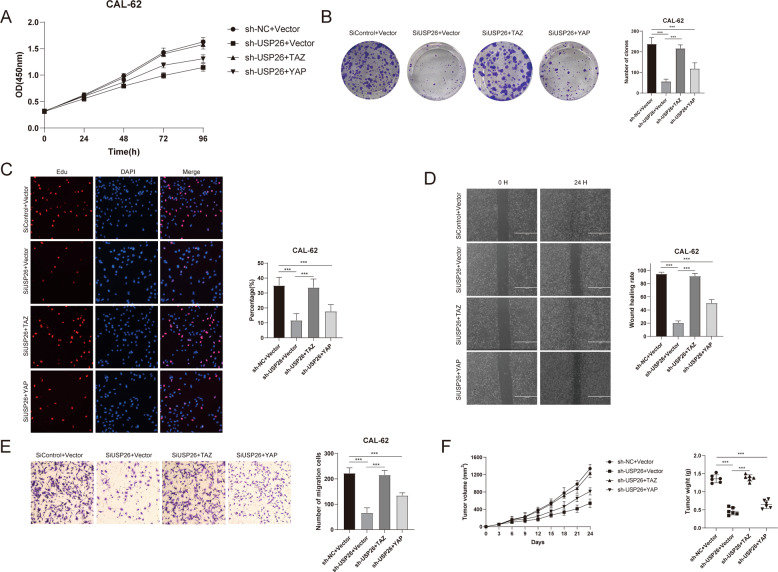
Increased TAZ expression reverses the effect of USP26 depletion. **A** Cell proliferation assay of CAL-62. **B** Clone formation assay of CAL-62. **C** Representative images of EdU assay of CAL-62. **D** Wound-healing assay of CAL-62. **E** Transwell invasion assay of CAL-62. **F** Overexpression of TAZ in USP26-knockdown cells recovered tumor growth in vivo. **G** Overexpression of TAZ in USP26-knockdown cells recovered tumor metastasis in vivo. *P value < 0.05; **P value < 0.01; ***P value < 0.001.

We further investigated the function of USP26 in two ATC cell lines (CAL-62 and KHM-5M). USP26 depletion significantly decreased cell proliferation rate and induced G1/S arrest (Fig. 5A, B). Depletion of USP26 also corresponded to a reduction in clonogenicity (Fig. 5C). In agreement, EdU incorporation assay indicated that DNA synthesis was inhibited by the knockdown of USP26, (Fig. 5D, E). Furthermore, the knockdown of USP26 could also reduce the migration and invasion capacity of ATC cells as evaluated by wound healing and transwell invasion assays (Fig. 5F, G). The in vivo tumor growth assay showed that USP26 depletion significantly inhibited tumor growth in xenograft mice models (Fig. 5H, I). The in vivo metastatic assay showed that USP26 depletion could inhibit lung metastasis in mice. To determine whether the functions of USP26 in regulating cell proliferation, migration, and invasion through the effects of TAZ, we performed rescue experiments by overexpressing TAZ in USP26 knockdown cells. Increased TAZ expression induced cell proliferation and clonogenicity (Fig. 6A–C). Wound healing and transwell invasion assays indicated that the re-expression of TAZ largely rescued the migration and invasion capacity of ATC cells (Fig. 6D, E). We then examined the function of USP26 in vivo. The in vivo tumor growth assay indicated that the tumor growth was significantly decreased in USP26 depletion cells, which effect could be further abrogated by TAZ overexpression (Fig. 6F). We further used a tail vein injection mouse model for in vivo metastasis evaluation. Depletion of USP26, dramatically inhibited lung metastasis in mice, which effect could be rescued by TAZ overexpression (Fig. 6G). Taken together, these results indicate that USP26 promotes ATC progression through TAZ.

### USP26 and TAZ are uniformly overexpressed in thyroid cancer samples

**Fig. 7:**
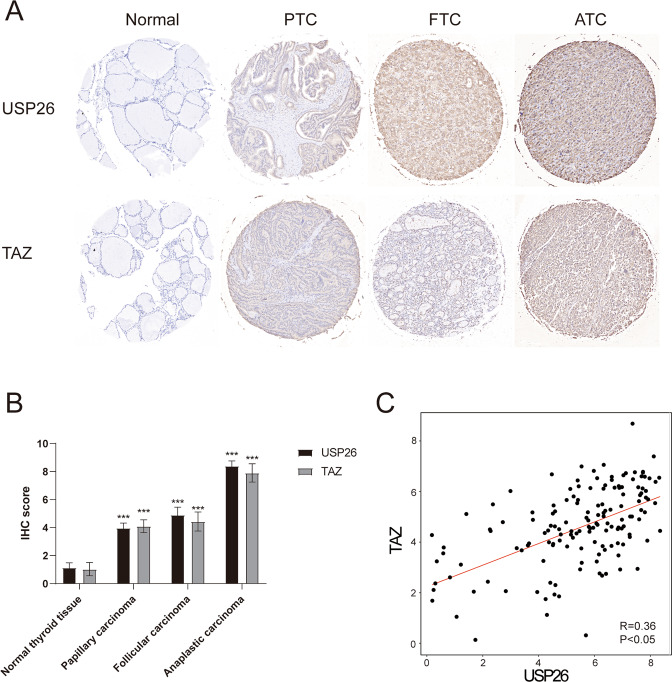
USP26 correlates with TAZ protein levels in human thyroid cancer samples. **A**, **B** USP26 and TAZ was upregulated in ATC. **C** USP26 correlated with TAZ in thyroid cancer samples. Commercially available tissue microarray slides (Alenabio, China.) were purchased for IHC analysis. Each microarray contained 140 samples, including normal thyroid tissue (*n* = 12), papillary thyroid carcinoma (*n* = 63), follicular carcinoma (*n* = 46), anaplastic carcinoma (*n* = 19).

To further study the relationship of USP26 and TAZ in thyroid cancer, we examined the expression of USP26 and TAZ in thyroid cancer tissues and normal tissues. Immunohistochemistry staining showed that the USP26 protein level was much higher in thyroid cancer samples than the normal tissues (Fig. 7A, B), indicating an oncogenic role of USP26 in thyroid cancer. In addition, there was a significant positive correlation between USP26 and TAZ protein level in both thyroid cancer tissues (Fig. 7C). This positive correlation further proved the regulating relationship between USP26 and TAZ.

## Discussion

ATC is considered as the most aggressive and lethal human malignancy [18]. Conventional therapies are unable to prolong the survival of patients with ATC, while recent improvement in understanding the genetic and molecular mechanisms of ATC hold promise for targeted therapy for this disease [3]. Ubiquitination is an important posttranslational modification, which is a central component of the cellular protein-degradation machinery and essential for cellular homeostasis [19]. The major part of ubiquitination process is mediated by three enzymes: ubiquitin-activating enzyme (E1), a ubiquitin conjugating enzyme (E2), and a ubiquitin ligase (E3) [20]. It should be noted that the ubiquitination of cellular proteins is a reversible and dynamic process, constantly being ubiquitinated and deubiquitinated. This process is precisely and orchestrated determined by several E3 ubiquitin ligases and DUBs [21–23]. The E3 ubiquitin ligases selectively mediate the ubiquitin conjugation of substrates, while DUBs negatively regulate this process [24]. Accumulating evidence has confirmed that DUBs play an important role in cancer progression. However, the potential roles of DUBs in ATC are largely unknown.

The Hippo signaling pathway is a novel and evolutionary conserved pathway, and has emerged as a critical signaling in regulating tumorigenesis. The transcriptional coactivators TAZ and YAP are the final transducer effectors of this pathway, which interact with TEA domain family transcription factors to activate the transcription of genes involved in various oncogenic activities, including cell growth, cell mobility, cell survival and metabolism [7, 25]. TAZ is overexpressed in various human malignancies, and play crucial roles in tumor initiation and progression [11, 26, 27]. Compare to YAP, TAZ is an extremely unstable protein which is primarily regulated by protein degradation [28]. PYK2 increases the tyrosine phosphorylation of LATS1/2 and TAZ through its tyrosine kinase activity, thus stabilizing TAZ and promoting TAZ-regulated cellular processes [25]. While phosphorylation of TAZ at Ser-311 leads to F-box protein β-TrCP-mediated ubiquitination and proteasomal degradation [29]. Interestingly, TRIB3 can interfere with the interaction between TAZ and β-TrCP and inhibit β-TrCP-induced degradation of TAZ [30]. USP1 acts as a post-translational modifier of TAZ, which directly interacts with and deubiquitinates TAZ [31]. In the present study, we fund higher expression of TAZ in thyroid cancer tissues compared to normal thyroid tissues. In the further analysis of the correlation between TAZ expression and clinicopathologic features, we observed that the expression of TAZ was related to tumor size and positively associated with tumor differentiation. While the mechanisms responsible for regulating TAZ expression in ATC remain largely unclear.

To identify the DUBs that can potentially deubiquitinate and stabilize TAZ, we have screened 39 DUBs by monitoring the protein levels of TAZ. USP26 was observed to be the most potent in deubiquitinating and stabilizing TAZ as its depletion dramatically decreased TAZ protein level. USP26 is essential for androgen receptor hormone‐induced activation in spermatogenesis and is predominantly expressed in testis. USP26 gene mutations could play crucial role human male infertility [32–34]. Several studies have suggested that expression of USP26 is not limited to germ cells, it is also expressed in somatic cells and tumor cells. USP26 negatively regulates somatic cell-reprogramming process by preventing the degradation of CBX4 and CBX6 through the removal of K48-linked polyubiquitination [35]. USP26 is highly expressed in esophageal squamous cell carcinoma (ESCC), which acts as a specific deubiquitinase of Snail and promotes ESCC cell migration and invasion [36]. In the present study, we performed a series of experiments and identified USP26 as a potent DUB responsible for TAZ deubiquitination and stabilization in ATC. First, USP26 and TAZ directly interact. Co-IP assay indicated that the USP domain of USP26 interacted with the C-terminal region of TAZ, and GST- pulldown assay detected the direct interaction between USP26 and TAZ. Second, USP26 enhances TAZ protein stabilization in a DUB activity-dependent manner and decreases TAZ polyubiquitination. We observed that depletion of USP26 markedly decreased TAZ protein level, and the decrease of TAZ could be further reversed by overexpression of wild type USP26, but not its catalytically inactive mutant, or addition of the proteasome inhibitor MG132. Upon inhibition of protein synthesis by cycloheximide, the half-life of TAZ was shortened in cells depleted of USP26, but prolonged in cells overexpressing USP26. In vivo and in vitro ubiquitylation assays confirmed that USP26 directly removed the ubiquitin chain from TAZ. Interestingly, USP26 preferred to de-ubiquitylate K29- and K48-linked polyubiquitination on TAZ protein. As polyubiquitination through K48 of Ub generally results in proteasomal degradation, USP26 may maintain the stability of TAZ by removing the K48-linked ubiquitin chain from TAZ protein. Our results also indicated that USP26 has important roles during the progression of ATC, as USP26 depletion significantly reduced cell proliferation, migration and invasion. USP26 may execute its function in ATC mainly through the regulation of TAZ. Because the restoration of TAZ expression in ATC cells abolished the suppressive effects induced by USP26 depletion. Consistently, in vivo tumorigenesis and metastasis assay suggested that USP26 functions as a promotor of tumor growth and metastasis.

In conclusion, our study demonstrates that USP26 is a potent DUB responsible for TAZ and USP26 promotes ATC progression by deubiquitinating and stabilizing TAZ. Our findings provide new insight into the roles of USP26 in Hippo signaling pathway in ATC, modulating the activity of USP26 or regulating its gene expression level could be a promising strategy to treat ATC.

## Supplementary information

supplementary legend

Figure S1

Figure S2
