# Supplementary material for: USP26 promotes anaplastic thyroid cancer progression by stabilizing TAZ
Source: Cell Death Dis. 2022 Apr 9;13(4):326. doi: 10.1038/s41419-022-04781-1 (PMC8994751; doi:10.1038/s41419-022-04781-1)
Supplement: Supplementary file 1 — supplementary legend [file 41419_2022_4781_MOESM1_ESM.docx]

**Supplementary legend**

**Figure S1.** (A). Interaction of USP26 proteins with wild type/constitutively active TAZ in 293T cells was analyzed by co-IP assay. (B). HA-Ub was co-transfected with Flag-TAZ (wild type or constitutively active form) and Myc-USP26 into HEK293 cells. After treatment with 10 μM MG132 for 6 h, cell lysates were subjected to ubiquitination assay and the ubiquitination level of TAZ was detected by HA antibody.

**Figure S2.** Wild-type USP26, but not USP26^C304S^ possesses DUB activity towards polyubiquitinated TAZ *in vitro.* Ubiquitinated TAZ was purified from HEK293 cells transfected with HA-Ub, and Flag-TAZ plasmids using anti-Flag affinity purification method. The ubiquitinated TAZ was incubated with GST-tagged wildtype USP26, and USP26^C304S^ proteins purified from bacteria in 20 µl reaction buffer containing at 37 °C for 2 h. The reactions were stopped by boiling in 1X SDS sample buffer and analyzed using IB with anti-HA and anti-GST antibodies.
