# Supplementary figures and images for: USP26 promotes anaplastic thyroid cancer progression by stabilizing TAZ
Source: Cell Death Dis. 2022 Apr 9;13(4):326. doi: 10.1038/s41419-022-04781-1 (PMC8994751; doi:10.1038/s41419-022-04781-1)

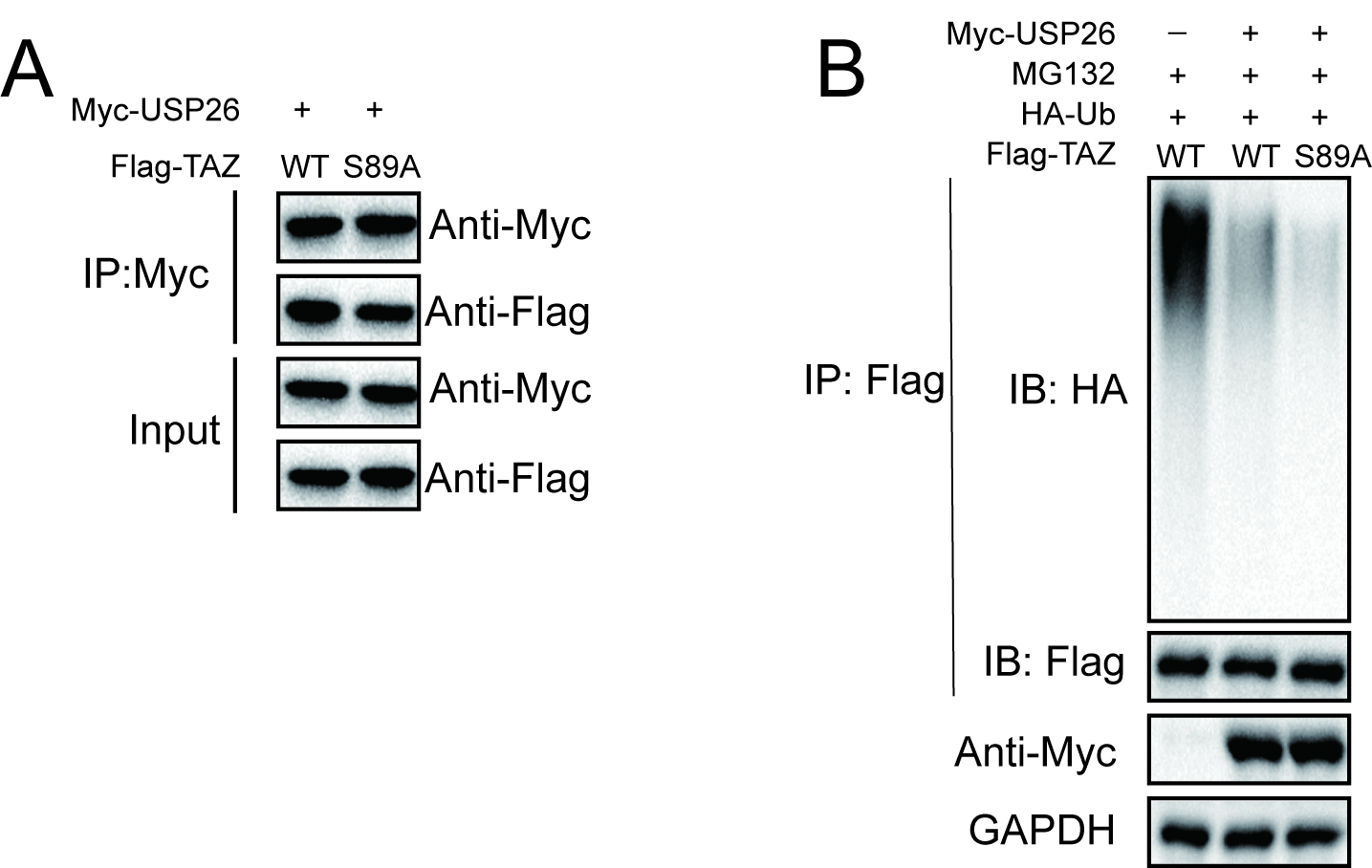

Supplement: Supplementary file 2 — Figure S1 [file 41419_2022_4781_MOESM2_ESM.tif]

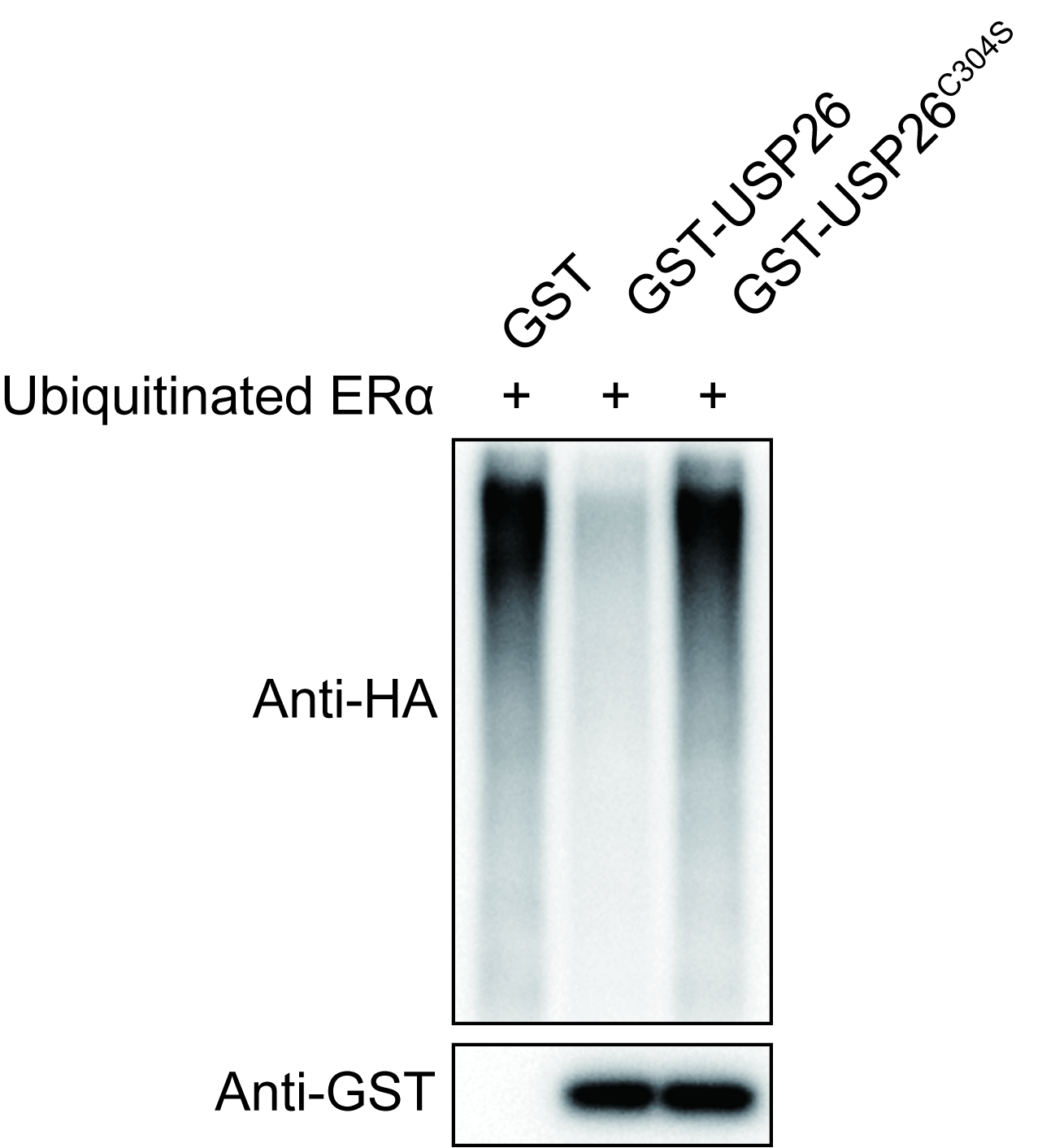

Supplement: Supplementary file 3 — Figure S2 [file 41419_2022_4781_MOESM3_ESM.tif]
